# Supplementary material for: In vivo multiomic Perturb-seq with enhanced nuclear gRNA capture
Source: bioRxiv. 2026 Mar 17:2026.03.15.711739. Preprint. [Version 1] doi: 10.64898/2026.03.15.711739 (PMC13015550; doi:10.64898/2026.03.15.711739)

bioRxiv preprint doi: https://doi.org/10.64898/2026.03.15.711739; this version posted March 17, 2026. The copyright holder for this preprint (which was not certified by peer review) is the author/funder, who has granted bioRxiv a license to display the preprint in perpetuity. It is made available under aCC-BY 4.0 International license.

Supplementary Figures

Supplementary Figure 1. Bead modification, nuclei capture and library construction workflow for gRNA recovery in sn-multiome profiling.

(a) Schematics of bead modification to introduce a gRNA-specific capture sequence onto BD Rhapsody capture beads. P in the circle indicates 5' phosphorylation on an oligonucleotide. (b) Schematics of nucleus capture on modified beads, showing recovery of gRNA-derived transcripts together with cellular transcripts and tagmented genomic DNA fragments. (c) Nested target-enrichment (TE) PCR workflow for gRNA library construction and sequencing configuration.

Supplementary Figure 2. *In vitro* benchmarking of gRNA recovery across vector constructs, bead modification rates, and amplification approaches.

(a) A UMAP visualization of nuclei profiled *in vitro* at different modification rates, colored by Seurat cluster and gRNA assignment. (b) Violin plots showing RNA quality-control metrics across nuclei in the *in vitro* dataset. (c) Single-gRNA assignment rates across the vector constructs at different bead modification rates. (d) gRNA assignment rates across bead modification rates and amplification approaches.

Supplementary Figure 3. Quality assessment of the *in vivo* sn-multiome dataset.

(a) Representative Bioanalyzer traces showing fragment size distributions of RNA, ATAC and gRNA libraries. (b) Violin plots showing normalized expression of representative marker genes across annotated cell types. (c-d) Violin plots showing RNA and ATAC quality-control metrics across nuclei profiled *in vivo*. nFeature_RNA: number of detected gene features per nucleus. nCount_RNA: total RNA UMI counts per nucleus. percent.mito: mitochondrial transcript fraction. nFeature_ATAC: number of accessible peaks detected per nucleus. nCount_ATAC: number of fragments per nucleus. TSS.enrichment: transcription start site enrichment score. FRiP: fraction of fragments in peaks. blacklist.ratio: fraction of ATAC molecules overlapping blacklist regions.

Supplementary Figure 4. Assessment of *in vivo* gRNA assignment rate and quality.

(a) gRNA assignment rates across cell types at different bead modification rates. (b) gRNA assignments across vector constructs show weak cross-gRNA correlations across bead modification rates. (c) Low cross-gRNA correlation between different gRNAs within the same animal and across animals. (d) The *in vivo* multiome Perturb-seq dataset generated in this study shows gRNA assignment rates comparable to those reported in other studies[9, 14] with only the transcriptome. (e) gRNA assignment rates across bead modification rates and amplification approaches. (f) Dot plot showing insertion/deletion frequencies and read depth at gRNA target loci from the multiome dataset across gRNA assignments, scaled by the maximum value in each row.

Supplementary Figure 5. Expression of 16 NDD risk genes.

(a-b) Dot plots showing expression of the 16 NDD risk genes across brain cell types (a) and developmental stages (b).

bioRxiv preprint doi: https://doi.org/10.64898/2026.03.15.711739; this version posted March 17, 2026. The copyright holder for this preprint (which was not certified by peer review) is the author/funder, who has granted bioRxiv a license to display the preprint in perpetuity. It is made available under aCC-BY 4.0 International license.

Supplementary Figure 6. gRNA distribution in the pooled AAV library and validation of sparse AAV labeling using low MOI.
(a) Distribution of gRNA pairs in the pooled AAV library determined by nanopore sequencing. Skew ratio defined as the 90th percentile divided by the 10th percentile of nonzero percentages. (b) Representative images of AAV-mediated labeling in the mouse cortex using three reporters, showing minimal co-transduction events. Yellow arrowheads indicate co-transduced cells labeled by more than one reporter (less than 1%). This viral titer at $1x10^9$ vg/embryo was optimal for maximizing the transduced cells while keeping the cells with multiple perturbations low. Scale bars 1mm and 100 μm. (c) FANS enrichment of GFP^+ nuclei following low MOI AAV delivery.

Supplementary Figure 7. Quality assessment of RNA, ATAC, and gRNA assignments in the *in vivo* multiome Perturb-seq screen.
(a) UMAP visualizations of RNA and ATAC modalities from the *in vivo* multiome dataset, colored by cell type. (b) gRNA assignment rates across annotated cell types. (c-d) Distribution of nuclei from the five samples across perturbations (c) and cell types (d). (e-f) Violin plots showing RNA and ATAC quality-control metrics across cell types (e) and animals (f), as described in Supplementary Figure 3c-d. (g) *Xist* expression in animals agreed with the sex of animals, supporting the animal identity assignment. M: male; F: female.

Supplementary Figure 8. Differential gene expression and chromatin accessibility analyses across perturbed cell types.
(a) Volcano plots of DE effects by *Sin3a* perturbations in Pvalb inhibitory neurons, L5 IT, L6 CT and L6 IT. (b) Scatter plot comparing DEGs by *Mef2c* perturbation in L6 IT to published RNA-seq dataset in *Mef2c* knockout mouse cortex[20]. The dark-gray line and light-gray shaded band show the linear regression fit and 95% confidence interval computed from shared genes, respectively. (c) Scatterplot comparing DEG results between L5 IT and L6 IT by *Sin3a* perturbation. The dark-gray line and light-gray shaded band show the linear regression fit and 95% confidence interval computed from all plotted genes, respectively. (d-e) Venn diagrams showing overlap of DEGs across Pvalb inhibitory neurons, L5 IT, L6 CT and L6 IT by *Mef2c* (d) and *Sin3a* (e). Color shading indicates the percentage of genes in each region relative to the total number of DEGs.

Supplementary Figure 9. Linked chromatin accessibility and gene expression changes at specific loci in *Mef2c*-perturbed L6 IT neurons revealed by joint RNA-ATAC profiling. (a-c) Representative loci showing linked chromatin accessibility and gene expression changes in *Mef2c*-perturbed L6 IT neurons. Asterisks mark significant DAR and DEG (P_{adj} <0.05).

Supplementary information
Table S1. Plasmids, gRNAs, oligonucleotides and AAV vectors.
Table S2. Sn-multiome library metadata and quality metrics.
Table S3. Sn-multiome perturbation phenotypic analysis summary.

bioRxiv preprint doi: https://doi.org/10.64898/2026.03.15.711739; this version posted March 17, 2026. The copyright holder for this preprint (which was not certified by peer review) is the author/funder, who has granted bioRxiv a license to display the preprint in perpetuity. It is made available under aCC-BY 4.0 International license.

Supplementary Figure 1

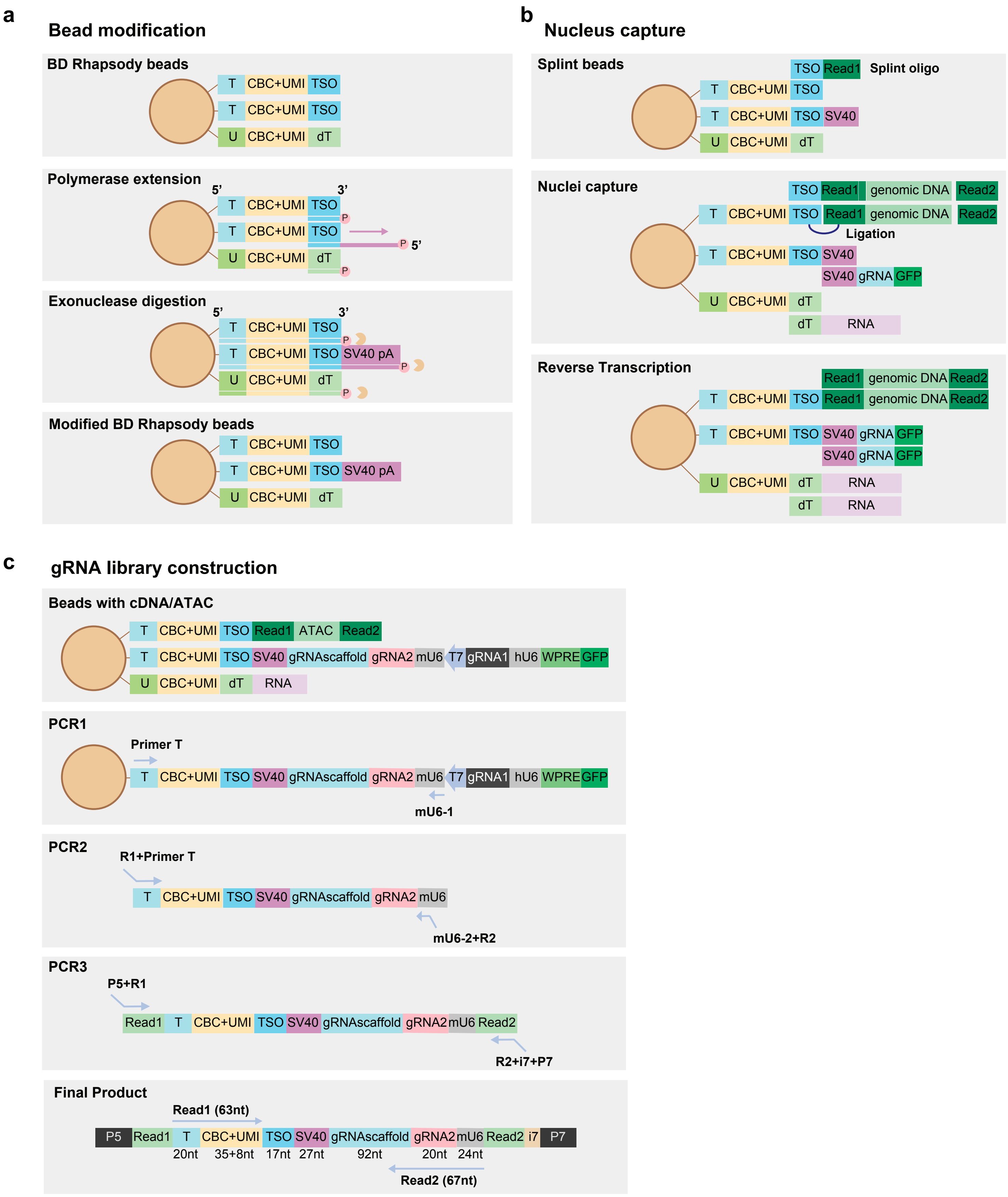

bioRxiv preprint doi: https://doi.org/10.64898/2026.03.15.711739; this version posted March 17, 2026. The copyright holder for this preprint (which was not certified by peer review) is the author/funder, who has granted bioRxiv a license to display the preprint in perpetuity. It is made available under aCC-BY 4.0 International license.

Supplementary Figure 2

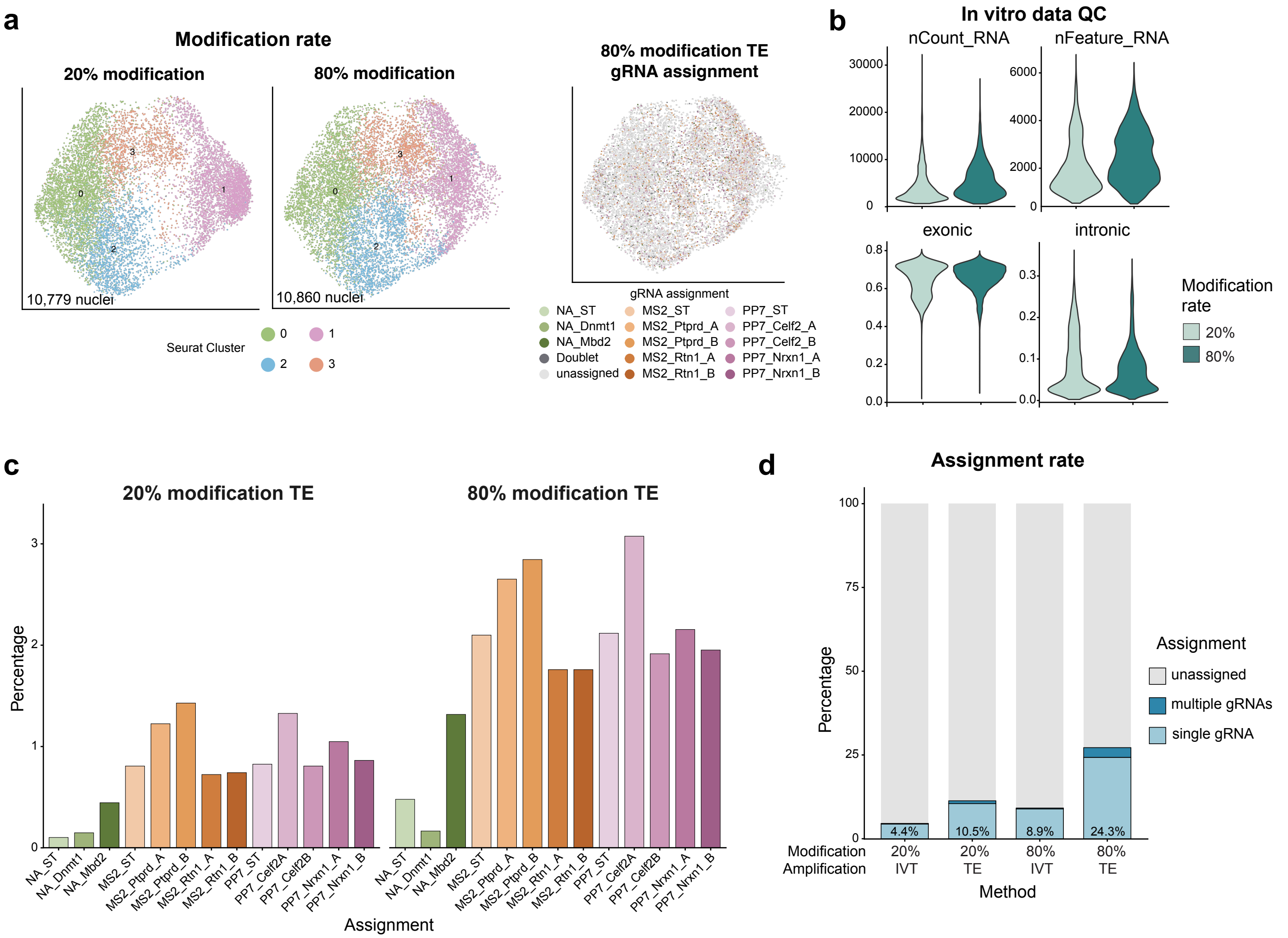

bioRxiv preprint doi: https://doi.org/10.64898/2026.03.15.711739; this version posted March 17, 2026. The copyright holder for this preprint (which was not certified by peer review) is the author/funder, who has granted bioRxiv a license to display the preprint in perpetuity. It is made available under aCC-BY 4.0 International license.

Supplementary Figure 3

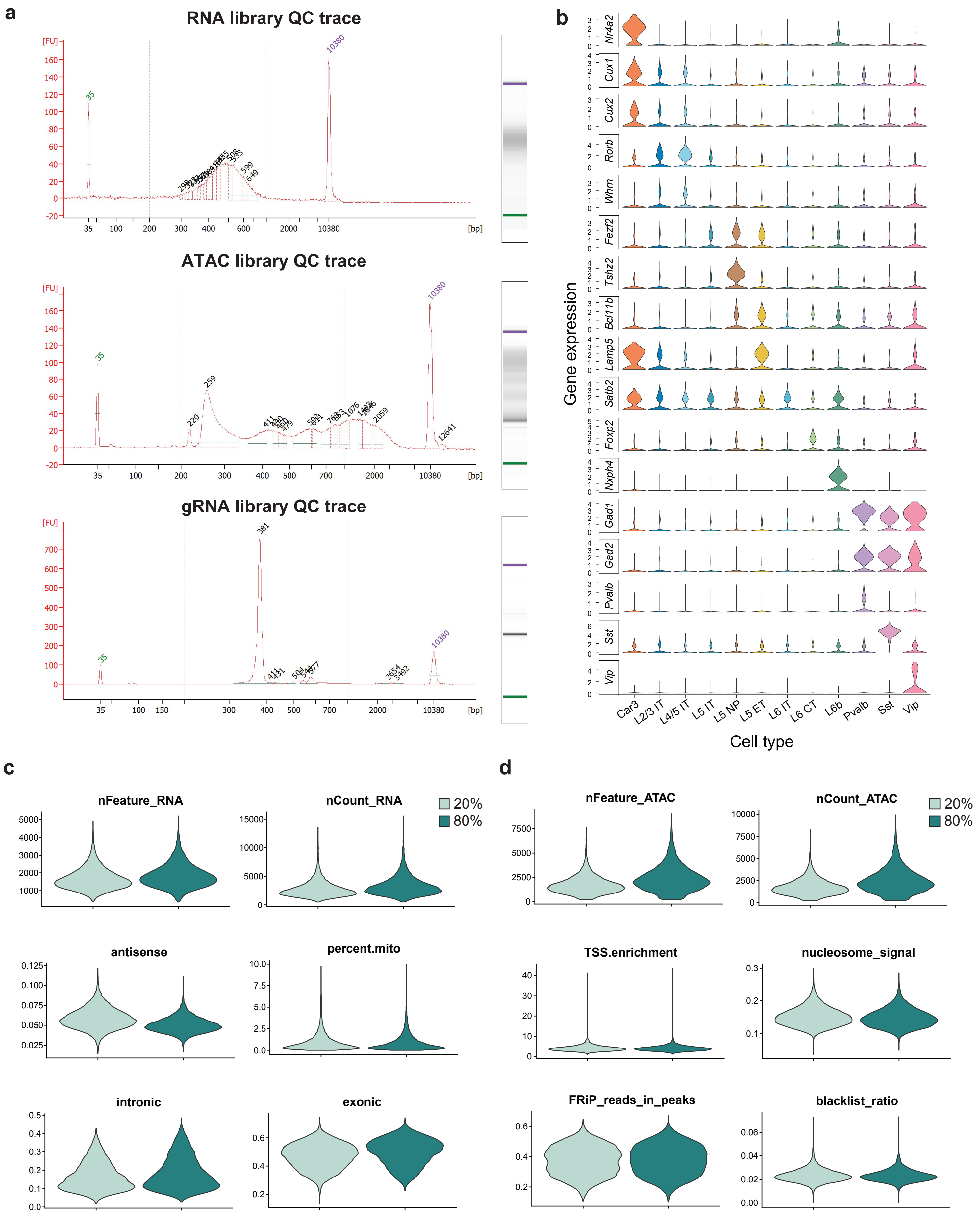

bioRxiv preprint doi: https://doi.org/10.64898/2026.03.15.711739; this version posted March 17, 2026. The copyright holder for this preprint (which was not certified by peer review) is the author/funder, who has granted bioRxiv a license to display the preprint in perpetuity. It is made available under aCC-BY 4.0 International license.

Supplementary Figure 4

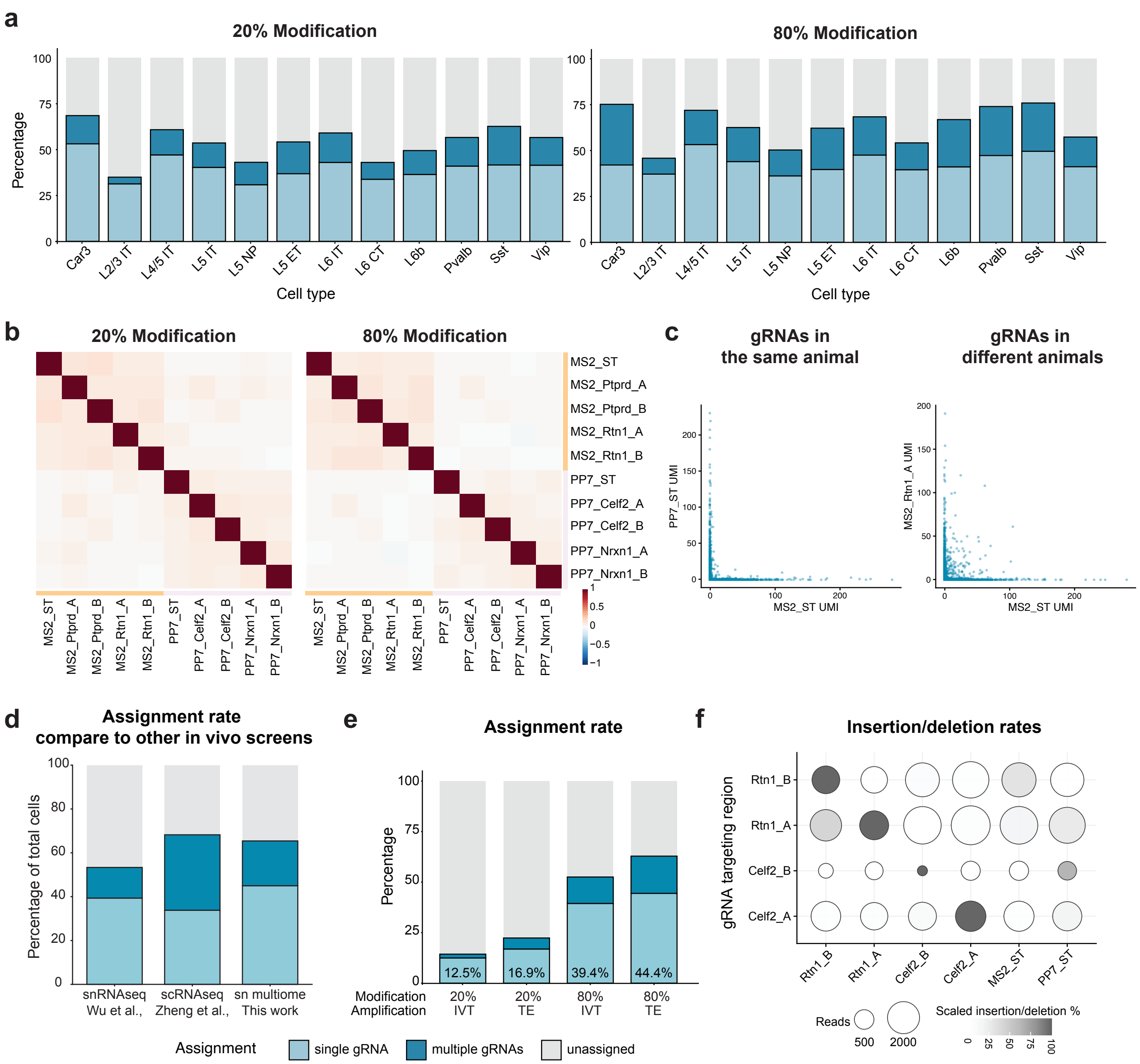

bioRxiv preprint doi: https://doi.org/10.64898/2026.03.15.711739; this version posted March 17, 2026. The copyright holder for this preprint (which was not certified by peer review) is the author/funder, who has granted bioRxiv a license to display the preprint in perpetuity. It is made available under aCC-BY 4.0 International license.

Supplementary Figure 5

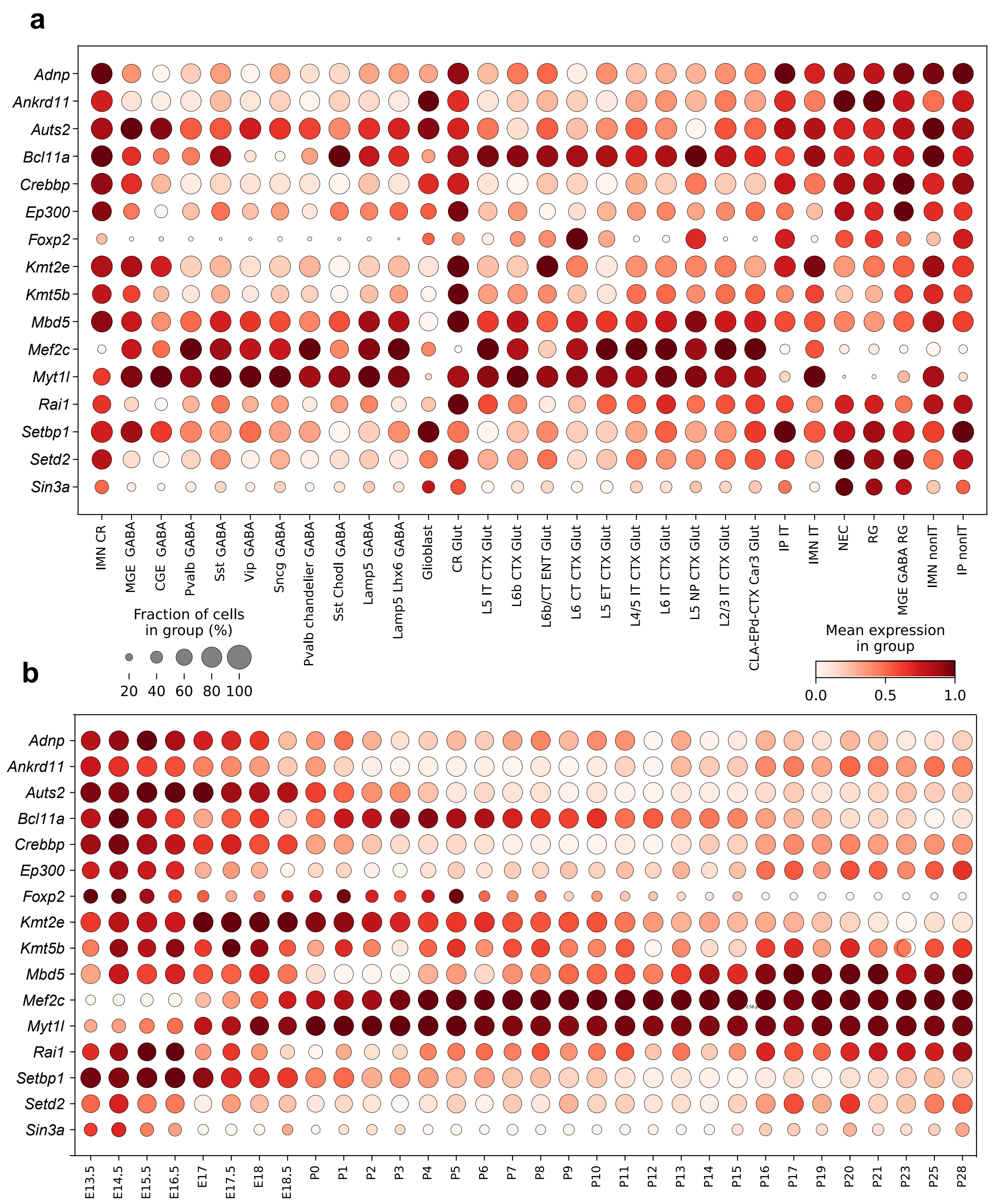

bioRxiv preprint doi: https://doi.org/10.64898/2026.03.15.711739; this version posted March 17, 2026. The copyright holder for this preprint (which was not certified by peer review) is the author/funder, who has granted bioRxiv a license to display the preprint in perpetuity. It is made available under aCC-BY 4.0 International license.

Supplementary Figure 6

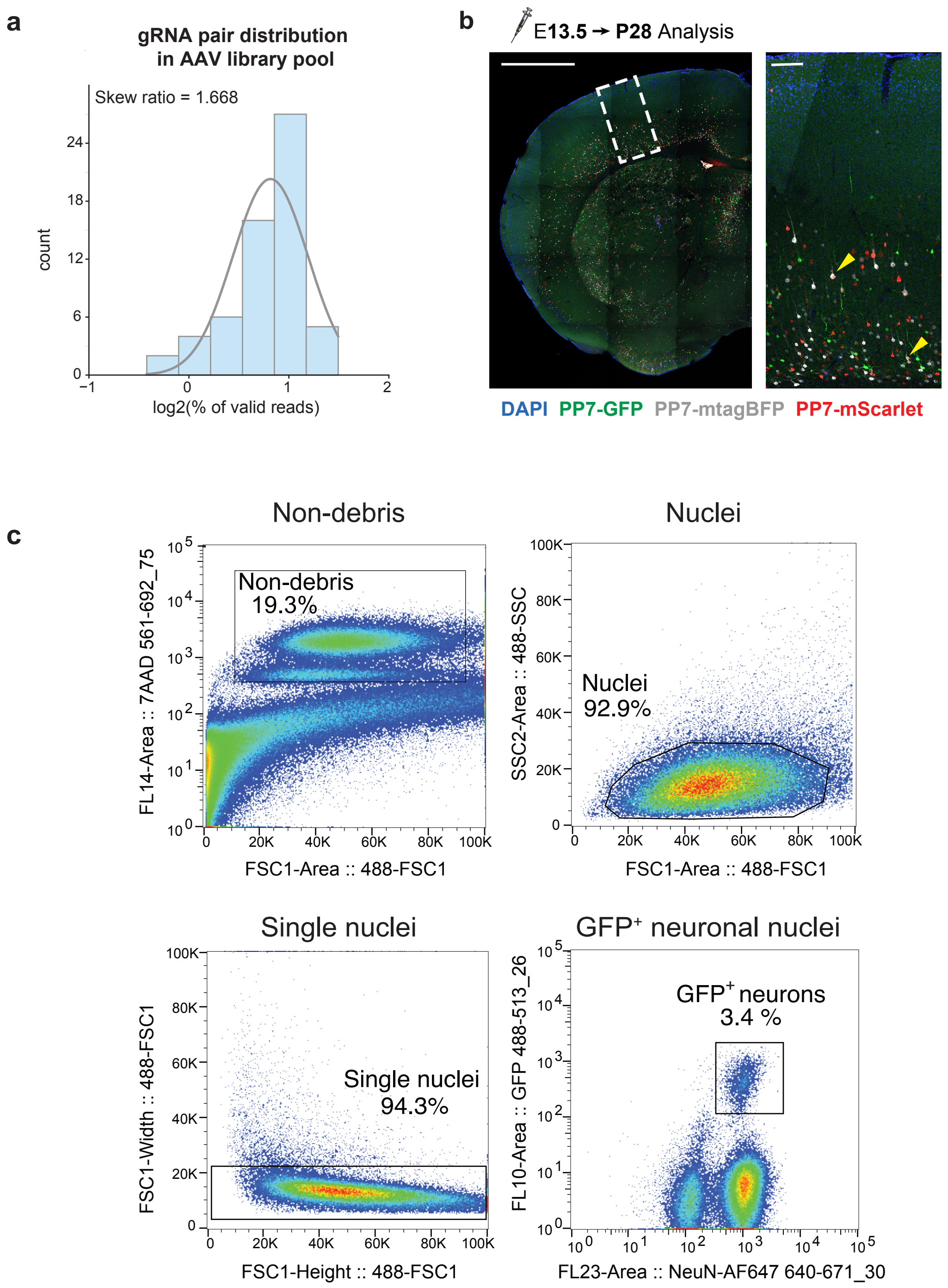

bioRxiv preprint doi: https://doi.org/10.64898/2026.03.15.711739; this version posted March 17, 2026. The copyright holder for this preprint (which was not certified by peer review) is the author/funder, who has granted bioRxiv a license to display the preprint in perpetuity. It is made available under aCC-BY 4.0 International license.

Supplementary Figure 7

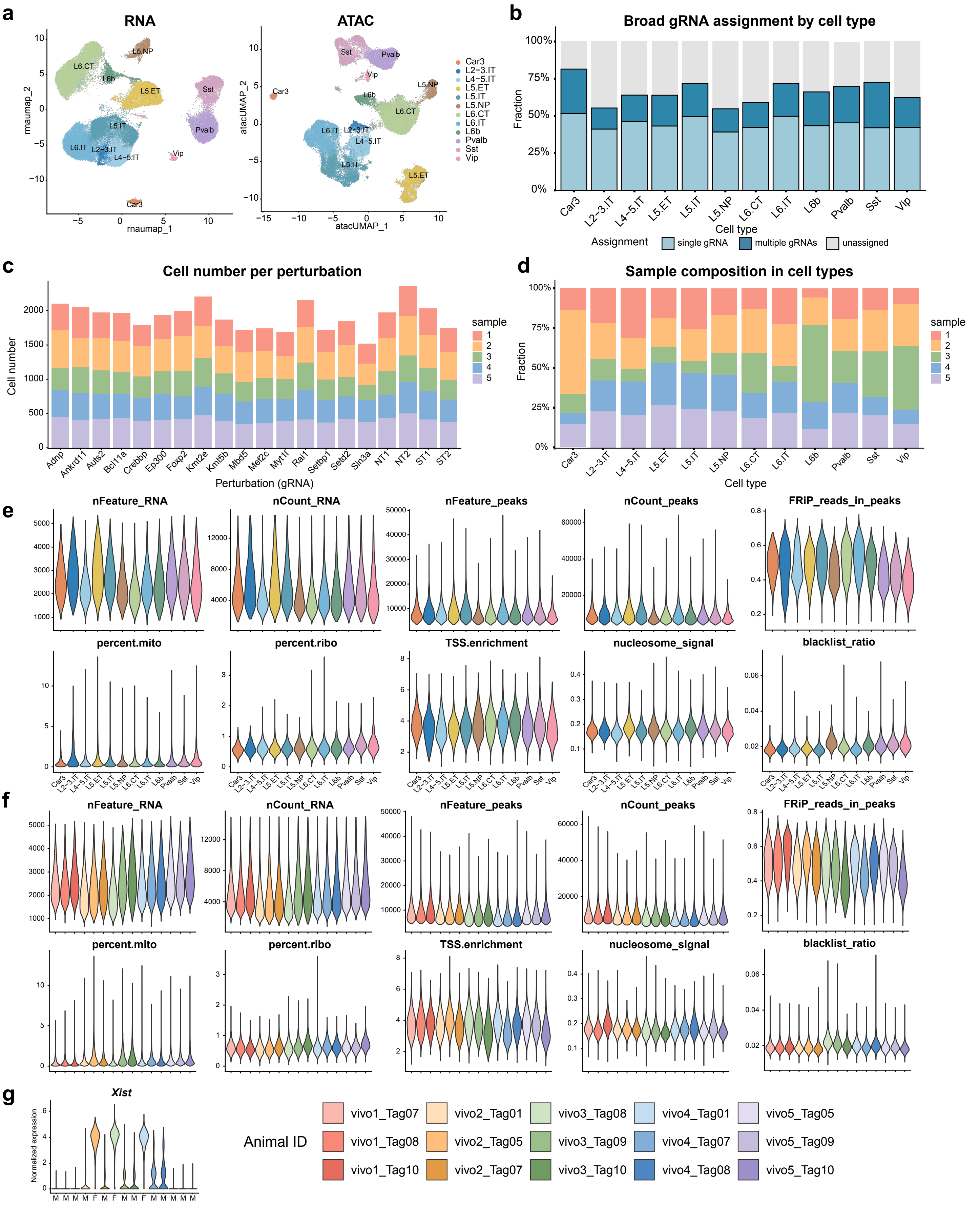

bioRxiv preprint doi: https://doi.org/10.64898/2026.03.15.711739; this version posted March 17, 2026. The copyright holder for this preprint (which was not certified by peer review) is the author/funder, who has granted bioRxiv a license to display the preprint in perpetuity. It is made available under aCC-BY 4.0 International license.

Supplementary Figure 8

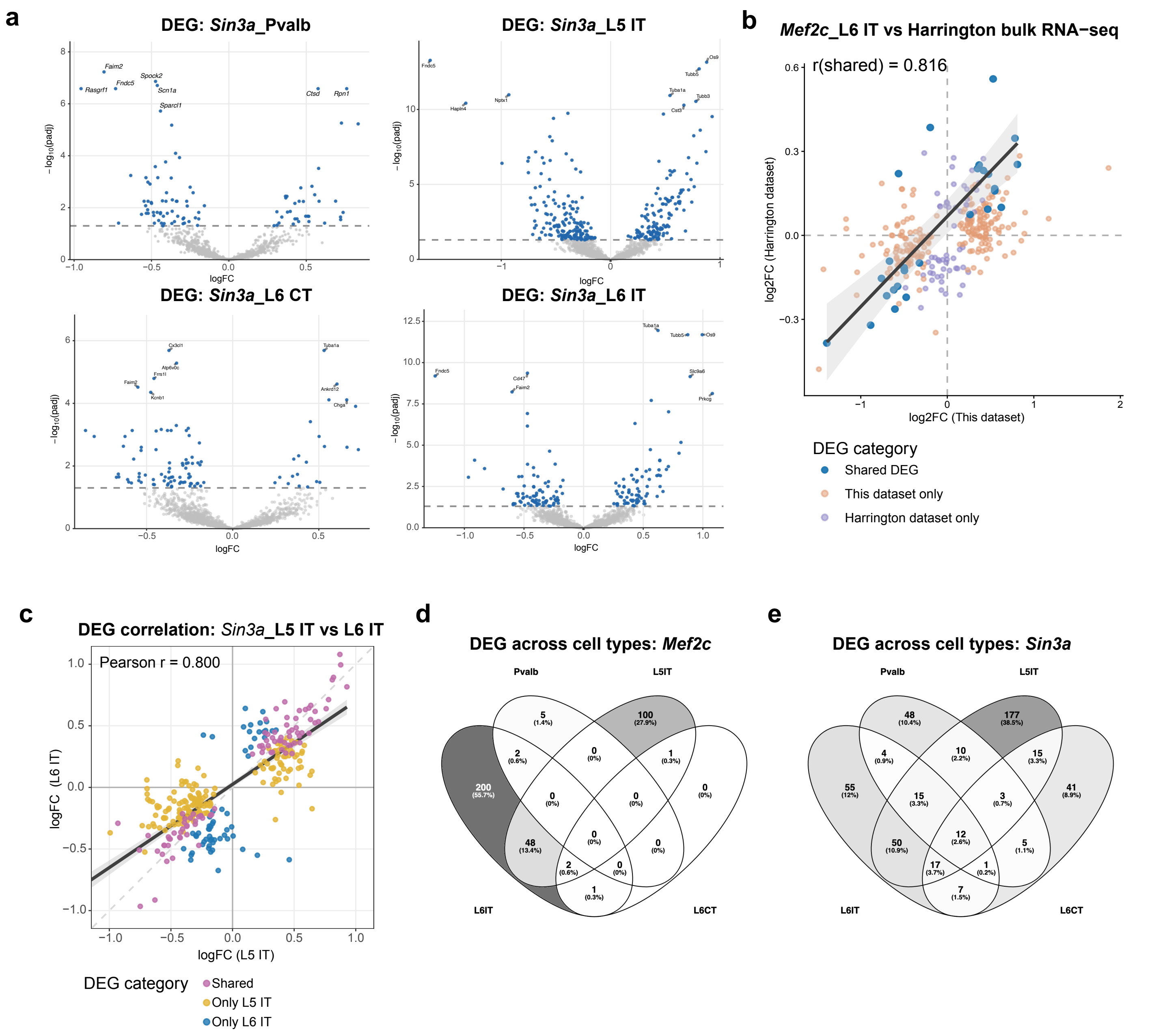

bioRxiv preprint doi: https://doi.org/10.64898/2026.03.15.711739; this version posted March 17, 2026. The copyright holder for this preprint (which was not certified by peer review) is the author/funder, who has granted bioRxiv a license to display the preprint in perpetuity. It is made available under aCC-BY 4.0 International license.

Supplementary Figure 9

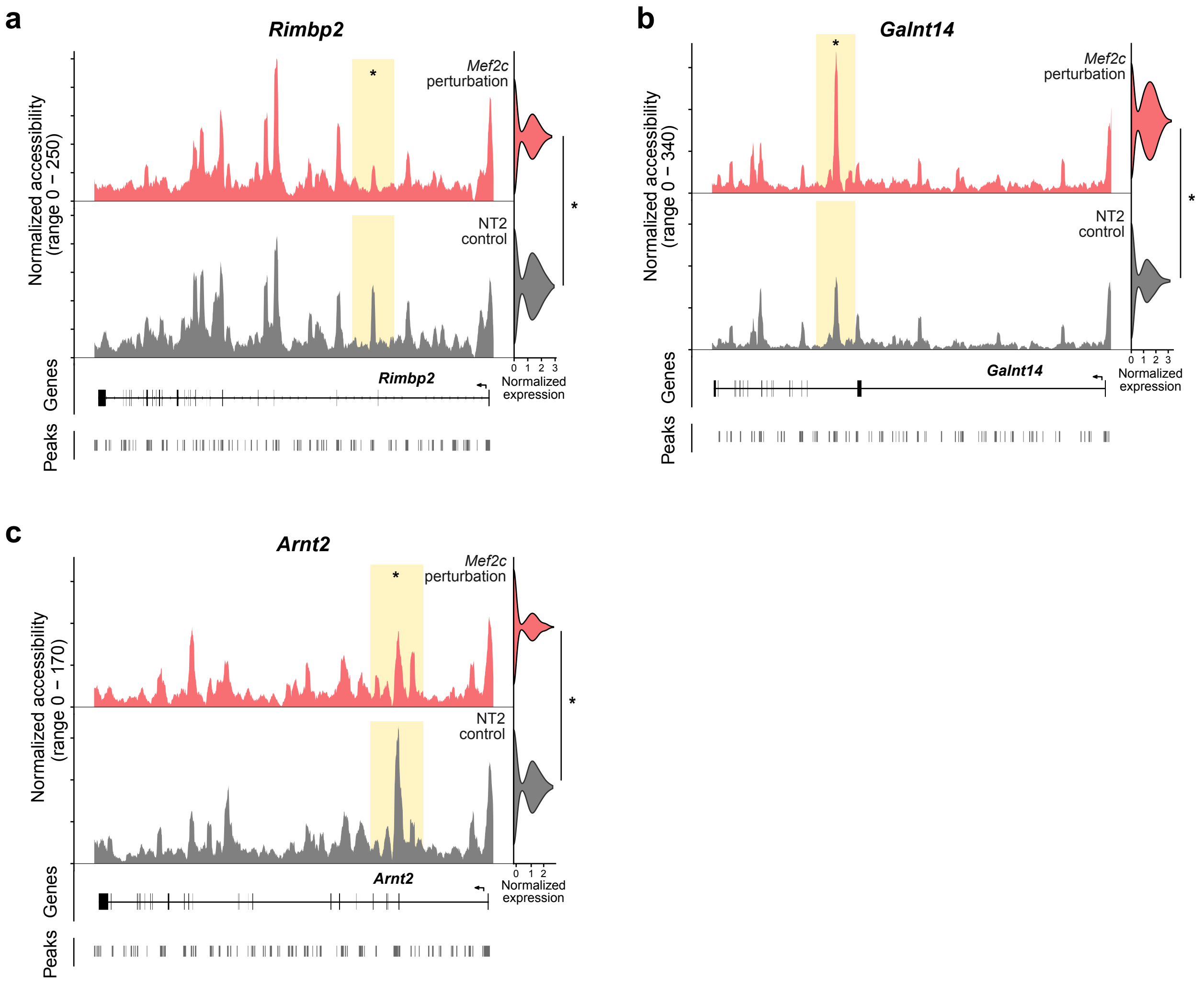

Supplement: Supplement 4 [file NIHPP2026.03.15.711739v1-supplement-4.pdf]
